# Coffee consumption and the risk of gastric cancer: a meta-analysis of prospective cohort studies

**DOI:** 10.1186/s12885-015-1758-z

**Published:** 2015-10-19

**Authors:** Liqing Li, Yong Gan, Chunmei Wu, Xianguo Qu, Gang Sun, Zuxun Lu

**Affiliations:** 1 Department of Social Medicine and Health Management, School of Public Health, Tongji Medical College, Huazhong University of Science and Technology, No. 13 Hangkong Road, Wuhan, 430030 Hubei China; 2 School of Economics and Management, Jiangxi Science and Technology Normal University, Nanchang, Jiangxi China; 3 School of Health Management, Hangzhou Normal University, Hangzhou, Zhejiang China

**Keywords:** Coffee consumption, Gastric cancer, Meta-analysis, Cohort studies

## Abstract

**Background:**

Several observational studies suggest that coffee consumption may be associated with an increased risk of gastric cancer, but the results are inconsistent. We conducted a meta-analysis to evaluate the relationship of coffee consumption with gastric cancer risk and quantify the dose–response relationship between them.

**Methods:**

Relevant prospective studies were identified by a search of PubMed, Embase, and Web of Science to May 2015 and by reviewing the references of retrieved articles. Two independent reviewers extracted data and performed the quality assessment. A random-effects model was used to calculate the pooled risk estimates and 95 % confidence intervals (CI). The heterogeneity was assessed using the *I*^2^ statistic. Publication bias was assessed by using funnel plot, the Begg test and the Egger test.

**Results:**

Thirteen prospective cohort studies with 20 independent reports involving 3,368 patients with gastric cancer and 1,372,811 participants during a follow-up period ranging from 4.3–8 years were included. Compared with the lowest consumption level of coffee, the pooled relative risk (RR) was 1.13 (95 % CI: 0.94–1.35). The dose–response analysis indicated that, the RR of gastric cancer was 1.03 (95 % CI; 0.95–1.11) for per 3 cups/day of coffee consumption. Any nonlinear association of gastric cancer risk with coffee consumption was not found (*P* for nonlinearity = 0.68). Subgroup analyses indicated that the pooled RR for participants from the United States comparing the highest with the lowest coffee consumption was 1.36 (95 % CI, 1.06–1.75, *I*^*2*^ = 0 %). In addition, people with higher coffee consumption was associated with 25 % higher risk of gastric cancer in equal to or less than 10 years follow-up group (RR = 1.25; 95 % CI, 1.01–1.55, *I*^*2*^ = 0 %). Visual inspection of a funnel plot and the Begg’s and the Egger’s tests did not indicate evidence of publication bias.

**Conclusions:**

This meta-analysis does not support the hypothesis that coffee consumption is associated with the risk of gastric cancer. The increased risk of gastric cancer for participants from the United States and equal to or less than 10 years follow-up group associated with coffee consumption warrant further studies.

## Background

Gastric cancer is the fourth most common cancer, behind lung, breast and colorectal cancers, and the second most common cause of cancer death in the world [1, 2]. It is estimated that 951,600 new stomach cancer cases and 723,100 deaths occurred in 2012. Gastric cancer rates are generally about twice as high in men as in women and vary widely among countries. Generally, the incidence of gastric cancer is highest in Eastern Asia (particularly in Korea, Mongolia, Japan, and China) [1]. Regional variations maybe reflect the differences in food storage, the availability of fresh produce and the prevalence of Helicobacter pylori infection [3]. Therefore, the identification of modifiable risk factors for the prevention of gastric cancer is of considerable public health importance. Besides Helicobacter pylori infection, smoking and alcohol intake, dietary factors are suggested to be associated with the development of gastric cancer [4–7].

Coffee is one of the most widely consumed beverages worldwide, with a yearly world average consumption of 1.1 kg per capita, which reaches 4.5 kg in industrialized countries [8]. Thus, any health effect of coffee is an important issue of public health [9]. More and more people and investigations focused on the association between coffee consumption and gastric cancer risk. The possible relation between coffee consumption and gastric cancer has been of considerable interest since the early 1960s, when a case–control study reported by Higginson suggested that the coffee might be a risk factor for gastric cancer [10]. Since then, a number of epidemiological studies have assessed the association between coffee consumption and gastric cancer risk, with the inconsistent results. A meta-analysis [11] in 2006 reported a null association between coffee consumption and gastric cancer risk, which took pooled effect size from 16 case–control studies and 7 cohort studies. Although the review included 7 cohort studies, the sample size was only 166,538, which lacked more powerful evidence. It is well known that prospective cohort study owned the strongest evidence in the observational studies. Prospective data to exclude some possible sources of bias that may exist in retrospective data could do good to come to more definitive conclusions [12]. The review did not fully explore the potential publication bias. Furthermore, the World Cancer Research Fund report of 2007 concluded that the evidence for an association between the consumption of coffee and the risk of gastric cancer was limited and inconsistent [13]. Since the publication of the last review on this topic, many more prospective studies have emerged, which could further contribute to the pooled data and allow further investigation into any association between coffee consumption and gastric cancer. Given that coffee is consumed very commonly and the morbidity and mortality of gastric cancer are high worldwide, clarifying this issue is of important public health and etiology implication. Thus, we performed an updated meta-analysis of prospective cohort studies to investigate the association between coffee consumption and the risk of gastric cancer and quantify the dose–response relationship of coffee consumption with gastric cancer risk.

## Methods

### Search strategy

This meta-analysis was conducted according to the checklist of the Meta-analysis of Observational Studies in Epidemiology (MOOSE) guidelines [14]. We comprehensively searched PubMed, Embase, and Web of Science databases from their inception through May 2015 for prospective cohort studies published in peer-reviewed journals describing an association between coffee consumption and risk of gastric cancer. We used “coffee” OR “caffeine” OR “decaffeinated” OR “dietary intake” OR “beverages” and “stomach” OR “gastric” combined with “cancer” OR “carcinoma” OR “tumor” “neoplasm” and “cohort studies” OR “prospective studies” OR “follow-up studies” as the search terms. The search was restricted to human studies. No restrictions were imposed on language. In addition, references of the retrieved articles were reviewed to identify additional studies. We did not contact authors of the primary studies for additional information.

### Inclusion criteria and exclusion criteria

Studies meeting the following criteria were included in the meta-analysis: (1) the study was a prospective cohort study design; (2) frequency and amount of coffee consumption were provided; (3) the exposures of interest were total coffee, caffeinated coffee, or decaffeinated coffee consumption; (4) the outcome of interest was gastric cancer; (5) the participants were free of gastric cancer at study entry; (6) the study provided the relative risk (RR) and the corresponding 95 % confidence interval (CI) for the association between coffee consumption and gastric cancer or sufficient data to calculate them.

Studies were excluded if: (1) the study was case–control or cross-sectional design; (2) the exposure was mixed beverage, in which the effect of coffee could not be separated; (3) only surrogate nutrients of coffee were reported; and (4) no categories of coffee intake were reported that could not allow for adequate classification of intake. If multiple published reports were from the same study cohort, only the most recent or informative one was included. Two reviewers (L.Q.L and Y.G) independently reviewed all studies by title or abstract or full text. Disagreements were resolved through consultation with the third reviewer (Z.X.L).

### Data extraction

We extracted the following information from studies included: name of the first author, year of publication, study location, characteristics of study population at baseline, duration of follow-up, method of exposure assessment, outcome measurements, number of cases, number of participants, RR or hazard ratio (HR) and corresponding 95 % CI for all categories of coffee consumption, and covariates adjusted in the multivariable analysis. We extracted risk estimates with the most adjustment (when available). For dose–response analysis, when studies reported the consumption in milliliters per day or week or month, we standardized all data into cups per day, using standard units of 125 ml for coffee consumption [15]. Data extraction was conducted independently by two authors (L.Q.L and Y.G). Interobserver agreement was assessed using Cohen kappa (κ) and any disagreements were resolved by discussion with the third author (Z.X.L.).

### Quality assessment

Two reviewers (L.Q.L and Y.G) independently performed the quality assessment by using the Newcastle-Ottawa Scale [16], which is a nine-point scale that allocated points based on the selection process of cohorts (0-4points), the comparability of cohorts (0–2 points), and the assessment of outcomes of study participants (0-3points). We assigned scores of 0–3, 4–6, and 7–9 for low, moderate, and high quality of studies, respectively.

### Statistical analyses

We preferentially pooled multivariable adjusted risk estimates where such estimates were reported. If adjusted analysis was unavailable (*n* = 3 studies), we pooled the unadjusted estimate. The RRs were considered as the common measurement of the association between coffee consumption and gastric cancer, and the HRs were considered equivalent to RRs. As different studies might report different exposure categories (dichotomous, thirds, quarters, or fifths), we used the study specific RR for the highest versus the lowest category of coffee consumption exposure for the meta-analysis. We pooled the RRs for the highest versus the lowest exposure categories of coffee consumption from each study using random-effects models, which consider both within- and between-study variation [17]. Any studies stratified by sex or type of gastric cancer were considered as independent reports.

We performed the dose–response meta-analysis based on the method described by Greenland and Longnecker [18] and Orsini et al. [19]. The amount of coffee consumption, the distributions of cases and person years, and RRs and 95 % CI were extracted according to the method. If the person years were not available for each category of coffee consumption, but reported the total number of cases/person-years, we estimated the distribution. If consumption of coffee was analyzed by quartiles (and could be approximated), e.g., the total number of person years was divided by 4 when the data were analyzed by quartiles in order to derive the number of person-years in each quartile [20].

The median or mean coffee consumption in each category was assigned to the corresponding dose of consumption. The midpoint of the upper and lower boundaries was considered the dose of each category if the median or the mean intake per category was not available. If the highest category was open-ended, the midpoint of the category was set at 1.5 times the lower boundary. When the lower boundary for the lowest category was not provided, the assigned median value was half of the upper boundary of that category.

To evaluate a potential non-linear dose–response relationship between coffee consumption and the risk of gastric cancer, we used a restricted cubic spline regression model with three knots at percentiles 10 %, 50 %, and 90 % of the distribution [21]. A *P* value for nonlinearity was calculated by testing against the null hypothesis that the coefficient of the second spline transformation was equal to zero [22].

Statistical heterogeneity among studies was evaluated using the *I*^*2*^ statistic, where values of 25 %, 50 % and 75 % represent cut-off points for low, moderate and high degrees of heterogeneity, respectively [23]. Subgroup analyses for sex, ethnicity, age, smoking, alcohol intake, and body mass index (BMI) were conducted to explore potential sources of study heterogeneity and examine the robustness of the primary results. In sensitive analyses, we conducted a leave-one-out analysis [24] for each study to examine the magnitude of influence of each study on pooled RRs. Potential publication bias was evaluated through funnel plot and with the Begg’s and the Egger’s tests [25, 26]. All analyses were performed with STATA statistical software (version 12.0; College Station, TX, USA). All tests were two sided with a significance level of 0.05.

## Results

### Literature search and study evaluation

**Fig. 1:**
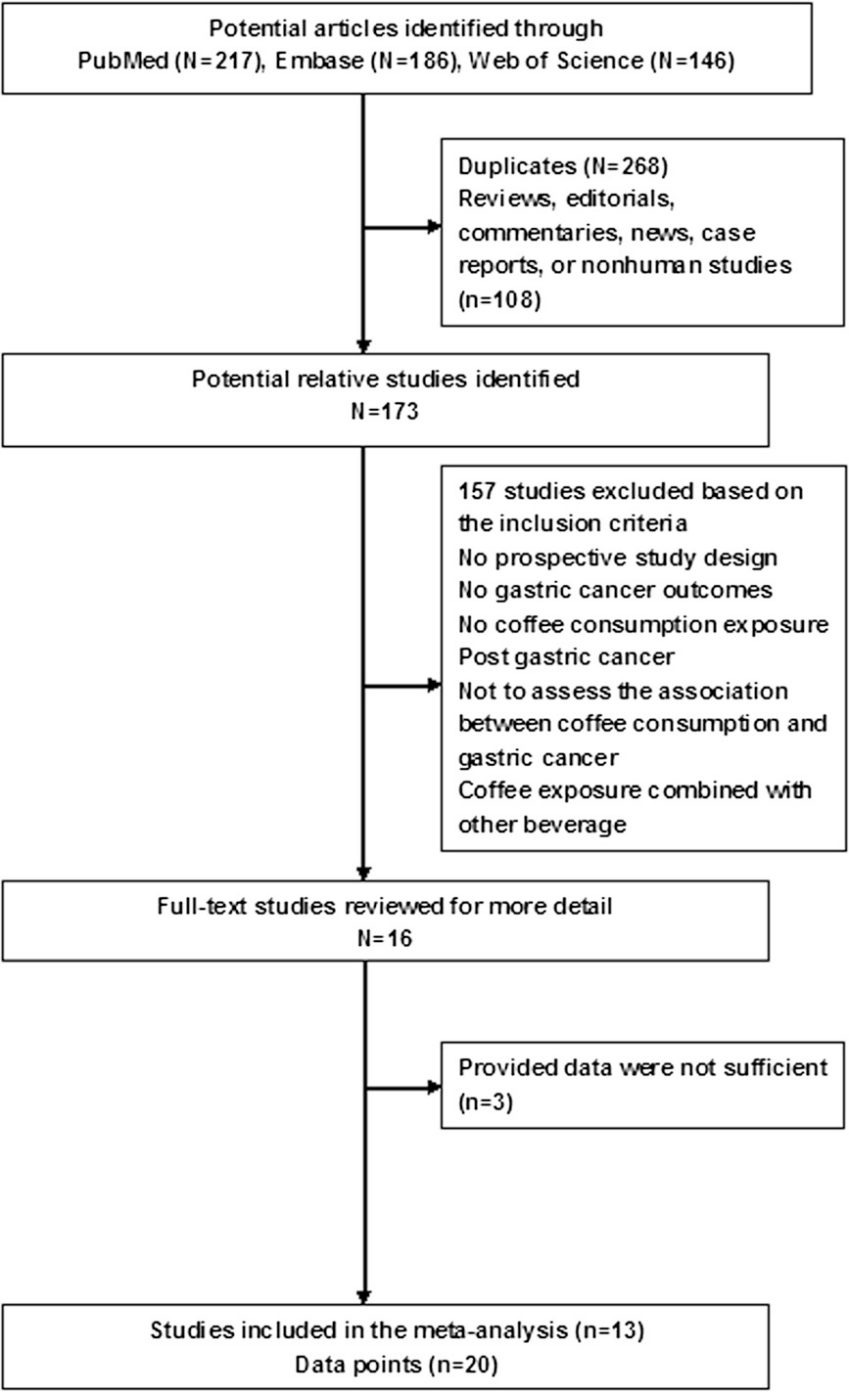
Flow chart showing the relevant studies of coffee consumption in relation to gastric cancer

The process of study identification and inclusion was shown in Fig. 1. Initially we retrieved 217 articles from the PubMed, 186 articles from the Embase, and 146 articles from the Web of Science. Of which 173 articles were identified as potentially relevant. After assessing the titles and abstracts, 157 studies were excluded because of non-compliance with the inclusion criteria. After retrieving the full text review of the remaining 16 articles for detailed evaluation, 3 articles were excluded because they did not report RRs and the corresponding 95 % CI of interest or provide sufficient data to calculate them. Finally, 13 prospective cohort studies [27–39] were included in the meta-analysis. Interobserver agreement (κ) between reviewers for study inclusion was very high (κ = 0.98). The average score for the quality assessment of included studies was 7.8, and the score for all studies was 6 or above (moderate or high quality). Notably, in dose–response analysis, 2 studies [31, 33] were excluded because of less than three categories of coffee consumption, and 2 studies [32, 36] were excluded because either the number of case or person years of each coffee consumption category was not available. Finally, 9 studies [27–30, 34, 35, 37–39] were included in the dose–response analysis of coffee consumption with the risk of gastric cancer.

### Study characteristics

The characteristics of 13 prospective cohort studies included are summarized in Table 1. These studies were published between 1986 and 2015. The size of the cohorts ranged from 3,158–481,563, with a total 1,372,811and the follow-up duration ranged from 4.3–18 years. The number of gastric cancer cases diagnosed in the primary studies ranged from 51–683, with a total of 3,368. Three studies were conducted in the United States [28, 30, 36], two in Norway [27, 29], two in Japan [32, 33], two in Sweden [34, 35], one in Netherlands [31], one in Finland [37], and one in Singapore [38]. (The study of Sanikini et al. [39] was a multi-country study conducted in Europe). Four studies [27, 32, 35, 36] reported results for both men and women, six studies [29, 30, 33, 37–39] reported the results by sex separately, one study [34] reported results for women only, and two studies [28, 31] reported results for men only. One study [36] reported results by anatomical site. Six studies [27, 28, 30, 31, 33, 37] assessed coffee consumption without using a specific dietary assessment method, and the rest of the studies assessed coffee consumption by food frequency questionnaires (FFQ) or diet records.

**Table 1:** Characteristics of studies included in the meta-analysis

Study source	Sex	Follow-up (years)	Age at baseline (years)	No of participants	No of case	Exposure assessment	Outcome assessment	Coffee consumption categories (highest vs. lowest)	Relative risk (95 % CI)	Adjustment for covariates	Study quality
Stensvold & Jacobsen, 1994, Norway	M	10.1	35–54	21,735	46	FFQ	Norwegian Cancer Registry, death certificates	≥7cups/d vs. ≤ 2cups/d	0.68 (0.28–1.69)	No covariate adjustment	7
Stensvold & Jacobsen, 1994, Norway	F	10.1	35–54	21,238	32	FFQ	Norwegian Cancer Registry, death certificates	≥7cups/d vs. ≤ 2cups/d	0.47 (0.16–1.39)	No covariate adjustment	7
Bidel et al., 2013, Finland	M	18	26–74	29,159	181	Self-administered questionnaire	Finnish Cancer Registry	≥10cups/d vs. ≤ 0cup/d	0.53 (0.26–1.09)	Age, study year, education, cigarette smoking, alcohol consumption, leisure time physical activity, history of diabetes, tea consumption, and BMI	9
Bidel et al., 2013, Finland	F	18	26–74	30,882	118	Self-administered questionnaire	Finnish Cancer Registry	≥10cups/d vs. ≤ 0cup/d	2.07 (0.53–8.15)	Age, study year, education, cigarette smoking, alcohol consumption, leisure time physical activity, history of diabetes, tea consumption, and BMI	9
Larsson et al., 2006, Sweden	F	15.7	39–73	61,433	160	FFQ	National and Regional Swedish Cancer registry, (ICD-9 codes)	≥4cups/d vs. ≤ 1cup/d	1.86 (1.07–3.25)	Age, time period, education, alcohol intake and tea consumption	7
Jacobsen et al.,1986, Norway	M/F	11.5	35+	16,555	147	Self-administered questionnaire	Cancer Registry of Norway and deaths records from the Central Bureau of Statistics Registry, ICD-7 codes	≥7cups/d vs. ≤ 2cups/d	1.32 (0.76–2.30)	Sex, age and residence	7
Nilsson et al., 2010, Sweden	M/F	6	40–60	64,603	70	FFQ	Regional cancer registry, ICD-7codes	≥4cups/d vs. < 1cup/d	0.99 (0.44–2.21)	Age, sex, BMI, smoking, education, and recreational physical activity	7
Khan et al., 2004, Japan	M	13.8	40–97	1,524	36	Self-administered questionnaire	Medical records, ICD-9 codes	Took several times per week + took every day vs. took never + took several times per year + took several times per month	1.00 (0.50–2.00)	Age, smoking	9
Khan et al., 2004, Japan	F	14.8	40–97	1,634	15	Self-administered questionnaire	Medical records, ICD-9 codes	Took several times per week + took every day vs. took never + took several times per year + took several times per month	0.30 (0.10–1.40)	Age, health status, health education, health screening & smoking	9
Tsubono et al., 2001, Japan	M/F	9	40+	26,311	419	FFQ	Miyagi Prefectural Cancer Registry records	≥3cups/d vs. never	1.00 (0.60–1.60)	Sex; age; type of health insurance; history of peptic ulcer; cigarette smoking; alcohol consumption; daily consumption of rice; tea and consumption of meat, green or yellow vegetables, pickled vegetables, other vegetables, fruits, and bean-paste soup	9
Galanis et al., 1998, United States	M	14.8	18+	5,610	64	Self-administered questionnaire	Hawaii Tumor Registry	≥2cups/d vs. none	2.20 (0.90–5.30)	Age, years of education, Japanese place of birth, smoking and alcohol intake	8
Galanis et al., 1998, United States	F	14.8	18+	6,297	44	Self-administered questionnaire	Hawaii Tumor Registry	≥2cups/d vs. none	1.60 (0.70–3.80)	Age, years of education, Japanese place of birth, and smoking	8
Nomura et al., 1986, United States	M	15	45–68	7,355	106	Interview	Hawaii Tumor Registry	≥5cups/d vs. none	1.18 (0.61–2.27)	Age	8
van Loon et al., 1998, Netherlands	M	4.3	55–69	58,279	146	Self- administered questionnaire	Regional cancer registries in the Netherlands and with a national pathology register	>4cups/d vs. ≤ 3cups/d	1.5 (0.95–2.36)	No covariate adjustment	6
Ren et al., 2010, United States	M/F	5.4	50–71	481,563	231	FFQ	State cancer registry databases, ICD-3 codes	Cardia ≥3cups/d vs. < 1cup/d	1.57 (1.03–2.39)	Age, sex, tobacco smoking, alcohol drinking, BMI, education, ethnicity, usual physical activity throughout the day, vigorous physical activity, and the daily intake of fruit, vegetables, red meat, white meat, and calorie	7
Ren et al., 2010, United States	M/F	5.4	50–71	481,563	223	FFQ	State cancer registry databases, ICD-3 codes	Non-cardia ≥3cups/d vs. < 1cup/d	1.06 (0.68–1.64)	Age, sex, tobacco smoking, alcohol drinking, BMI, education, ethnicity, usual physical activity throughout the day, vigorous physical activity, and the daily intake of fruit, vegetables, red meat, white meat, and calorie	7
Ainslie-Waldman et al., 2014, Singapore	M	14.7	45–74	27,293	394	FFQ	Singapore Cancer Registry and the Singapore Registry of Births and Deaths	≥4cups/d vs. never/monthly	1.06(0.48–2.32)	Age, interview year, dialect, education, cigarette smoking status, number of cigarettes smoked per day, years smoked, BMI, caffeine, and total energy intake	9
Ainslie-Waldman et al., 2014, Singapore	F	14.7	45–74	34,028	253	FFQ	Singapore Cancer Registry and the Singapore Registry of Births and Deaths	≥4cups/d vs. never/monthly	0.76(0.23–2.53)	Age, interview year, dialect, education, cigarette smoking status, number of cigarettes smoked per day, years smoked, BMI, caffeine, and total energy intake	9
Sanikini et al., 2015, International	M	11.6	25–70	308,021	395	FFQ, recall record	Regional and national mortality registries, ICD-10 codes	≥557 ml/d vs. never/< 131 ml/d	1.51(1.06–2.16)	Age, center, smoking, BMI, physical activity, education level, diabetes, alcohol consumption, intake of energy, fiber, vegetable, fruit, fish and red and processed meat	9
Sanikini et al., 2015, International	F	11.6	25–70	169,291	288	FFQ, recall record	Regional and national mortality registries, ICD-10 codes	≥557 ml/d vs. never/< 131 ml/d	0.72(0.47–1.08)	Age, center, smoking, BMI, physical activity, education level, diabetes, alcohol consumption, intake of energy, fiber, vegetable, fruit, fish and red and processed meat	9

Abbreviations: *BMI* body mass index, *FFQ* food frequency questionnaire, *F* female, *ICD* International Classification of Diseases, *M* male

### Coffee consumption and the risk of gastric cancer

**Fig. 2:**
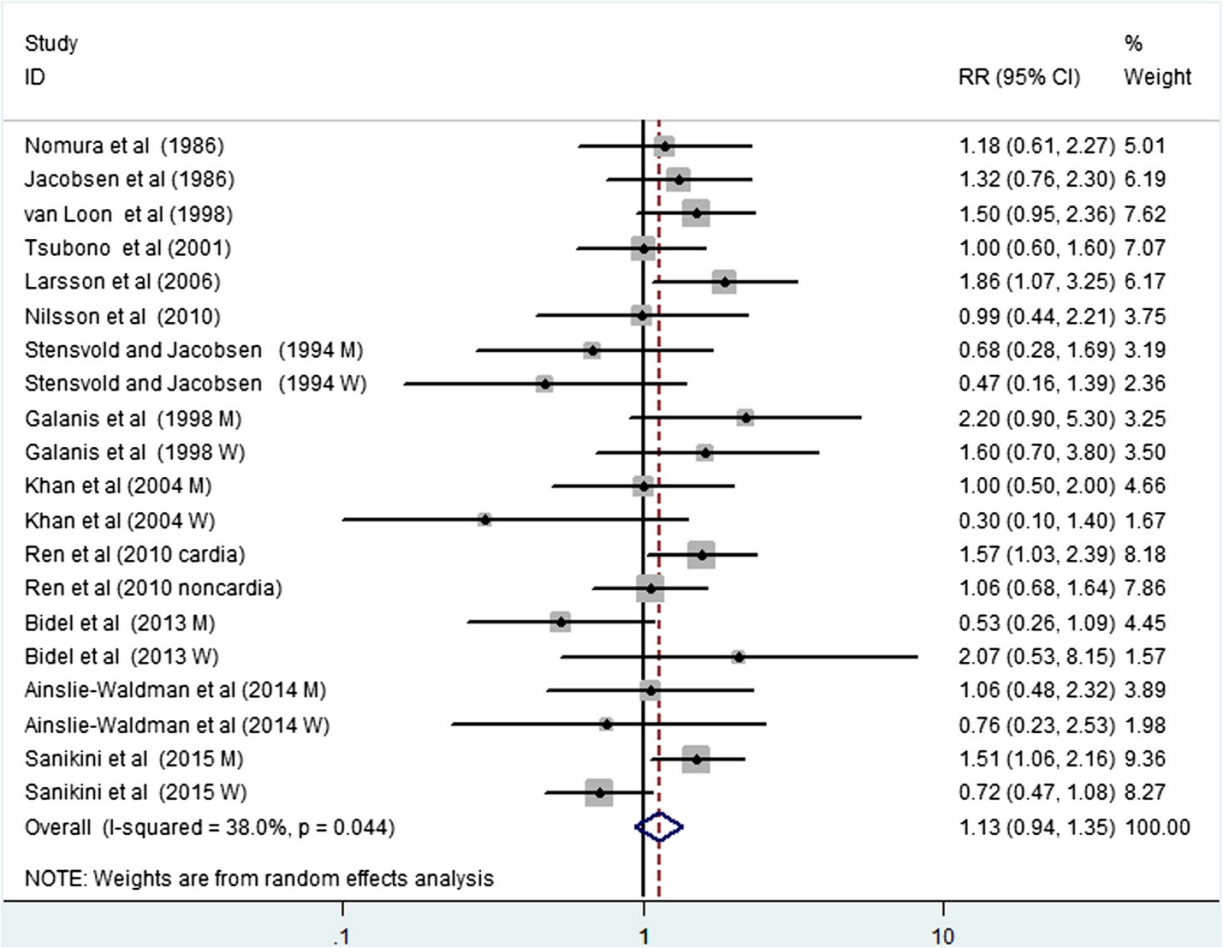
Forest plot of coffee consumption and the risk of gastric cancer

Figure 2 showed the results from the random-effects meta-analysis combining the RRs for gastric cancer in relation to coffee consumption. Eleven of 20 independent reports from 13 studies suggested a positive relation between coffee consumption and gastric cancer, while the other reports did not. Compared the lowest category of coffee consumption, the pooled RR of gastric cancer was 1.13 (95 % CI: 0.94–1.35) for the highest category of coffee consumption. A moderate heterogeneity was observed (*P* =0.044, *I*^2^ = 38 %).

### Dose–response analysis of coffee consumption with the risk of gastric cancer

**Fig. 3:**
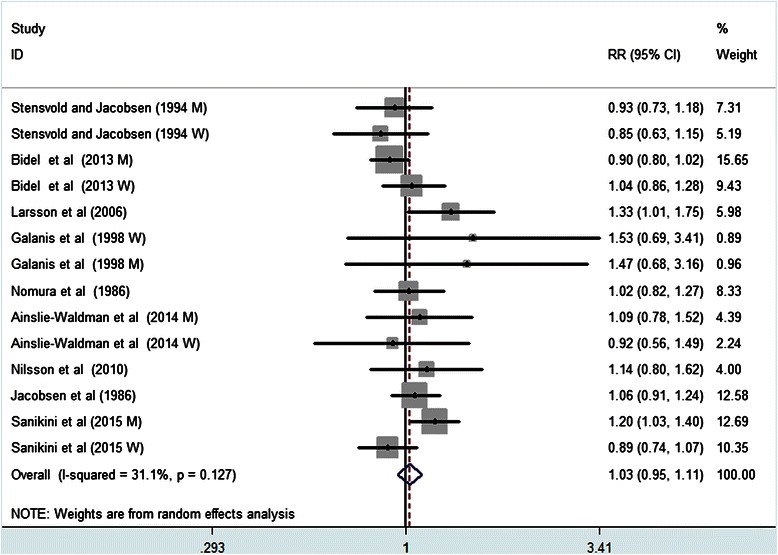
Risk of gastric cancer associated with per 3cups/day in coffee consumption

**Fig. 4:**
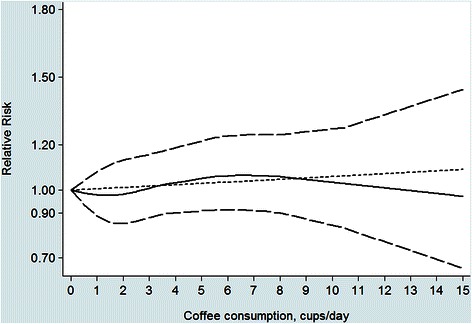
Dose–response relation plots between coffee consumption and the risk of gastric cancer

Nine studies with 14 reports were included in the dose–response analysis of coffee consumption and gastric cancer risk. The pooled estimate for the risk ratio per 3 cups/day increase in coffee was 1.03 (95 % CI, 0.95–1.11), with evidence of moderate heterogeneity (*I*^*2*^ = 31.1 %, *P* = 0.127) (Fig. 3). In the cubic spline model that included all studies, we did not find evidence suggesting any nonlinear association between coffee consumption and risk of gastric cancer (*P* for non-linearity = 0.68) (Fig. 4). Compared with people who had no daily consumption of coffee, the RR of gastric cancer estimated directly from the cubic spline model was 0.98(95 % CI; 0.89–1.08) for 1 cups per day, 0.98 (95 % CI; 0.85–1.13 for 2 cups per day, 1.06 (9 5% CI; 0.91–1.25) for 6 cups per day, and 1.06(95 % CI; 0.90–1.25) for 8 cups per day.

### Subgroup analyses

Subgroup analyses were conducted to examine the stability of the primary results and explore the resource of potential heterogeneity. No significant associations between coffee consumption and the risk of gastric cancer was identified in most subgroup analyses, which were stratified by sex, study quality, study location, follow-up duration, reference group, dietary assessment method (diet record/food frequency questionnaires versus other methods), and whether age, smoking, BMI, alcohol intake, tea consumption were controlled or not in models. However, a significant positive association between coffee consumption and gastric cancer risk was observed in the United States (RR = 1.36, 95 % CI, 1.06–1.75, *I*^*2*^ = 0.00 %, *P* = 0.536) and in the groups of equal to or less than 10 years follow-up (RR = 1.25, 95 % CI, 1.01–1.55, *I*^*2*^ = 0.00 %, *P* = 0.493) (see Table 2).

**Table 2:** Subgroup analyses of relative risk of gastric cancer

	No of reports	Relative risk	(95 % CI)	*I* ^*2*^	*P* for heterogeneity
Sex					
Men	8	1.17	0.88–1.55	37.10 %	0.133
Women	7	0.96	0.58–1.58	59.20 %	0.023
Combined	5	1.21	0.96–1.51	0.00 %	0.604
Study quality					
Score > 7	13	1.10	0.85–1.43	47.10 %	0.030
Score ≤ 7	7	1.20	0.94–1.53	20.10 %	0.276
Study location					
United States	5	1.36	1.06–1.75	0.00 %	0.536
Europe	10	1.08	0.80–1.45	56.80 %	0.013
Asia	5	0.92	0.66–1.28	0.00 %	0.532
Follow-up duration					
≤10 year	5	1.25	1.01–1.55	0.00 %	0.493
>10 year	15	1.06	0.82–1.37	47.10 %	0.022
Reference group					
None	8	1.10	0.81–1.49	17.30 %	0.294
Low consumption	12	1.13	0.89–1.42	49.60 %	0.026
Specific dietary assessment method					
Yes	11	1.10	0.87–1.38	41.50 %	0.072
No	9	1.17	0.85–1.61	40.30 %	0.099
Controlling age in models					
Yes	17	1.15	0.95–1.39	36.90 %	0.064
No	3	0.88	0.42–1.85	61.90 %	0.073
Controlling smoking in models					
Yes	13	1.06	0.84–1.33	43.80 %	0.046
No	7	1.29	0.97–1.71	21.80 %	0.263
Controlling BMI in models					
Yes	9	1.06	0.81–1.40	46.80 %	0.058
No	11	1.19	0.92–1.53	33.70 %	0.129
Controlling alcohol intake in models					
Yes	9	1.20	0.91–1.59	59.20 %	0.012
No	11	1.09	0.86–1.37	6.70 %	0.380
Controlling tea consumption in models					
Yes	6	1.09	0.74–1.62	67.60 %	0.009
No	14	1.18	0.97–1.43	13.00 %	0.310
Statistical model^*^					
Unadjusted	4	0.94	0.56–1.57	45.20 %	0.140
Adjusted	17	1.15	0.95–1.39	36.90 %	0.064

Abbreviations: *BMI* body mass index

*Study by Nilsson and colleagues reported results both statistical models for adjusted and unadjusted

### Sensitivity analyses

Sensitivity analyses were used to find potential origins of heterogeneity in the association between coffee consumption and gastric cancer, and to examine the influence of various exclusions on the combined RR, and check the robustness of all results above. Exclusion of two large sample studies [36, 39], whose size was more than half of total study samples showed a no statistically significant positive association1.11 (95 % CI, 0.89–1.38), and a medium heterogeneity was observed (*P* = 0.128, *I*^*2*^ = 29.6 %). We excluded any single study in turn and pooled the results of remaining studies, with a pooled RR of gastric cancer range from 1.09 (95 % CI, 0.90–1.32; *P* = 0.056)–1.19 (95 % CI, 0.99–1.41; *P* = 0.125), which indicated that none of the individual studies significantly influenced the overall result.

### Publication bias

**Fig. 5:**
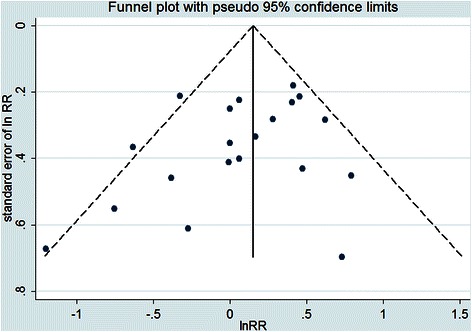
Funnel plot of coffee consumption and the risk of gastric cancer

Visual inspection of a funnel plot did not identify substantial asymmetry (see Fig. 5). The Begg rank correlation test and the Egger linear regression test also indicated no evidence of publication bias among the studies (Begg test *Z* = 1.27, *P* = 0.206; Egger test *t* = −1.42, *P* = 0.173).

## Discussion

This meta-analysis investigated the potential association between coffee consumption and gastric cancer risk, which based on 20 independent reports from 13 prospective cohort studies, involving 1,372,811 participants and 3,368 cases of new-onset gastric cancer. The results showed no appreciable overall association between coffee consumption and gastric cancer. Similar results were obtained in most subgroup analyses. Evidence of a nonlinear association of gastric cancer risk with coffee consumption was not observed.

### Comparison with previous studies

There were two meta-analyses published in 2014 [40] and in 2015 [41] to investigate the evidence about coffee consumption and gastric cancer. Results of this current meta-analysis generally concur and further complement the findings of previous review in several important aspects. In contrast to our results, Shen et al. found an overall positive association of 1.24 (95 % CI: 1.03–1.49) between the risk of gastric cancer and coffee consumption. One possible reason was that the review by Shen and colleagues only included eight studies involving 312,993 participants, and a few studies were missed, which may overestimate the effect size. Additionally, the review did not fully investigate other subgroups except for gender, follow-up time and ethnicity and conduct the dose–response analysis. Xie et al. indicated that no significant association was observed between coffee consumption and gastric cancer risk, which was consistent with our result. Compared with the review, our meta-analysis added one large scare cohort study with larger sample size and many more cases, which significantly enhanced statistical power to detect the potential associations of coffee consumption with gastric cancer. More importantly, the nonlinear association between coffee consumption and gastric cancer was investigated in present meta-analysis.

In the subgroup analysis, we obtained two valuable and important findings. A main finding was that the association between coffee consumption and the risk of gastric cancer was significant in the United States from three studies comprising 500,825 participants, but not in European and Japanese populations, which was a very interesting phenomenon. According to the international statistic of U.S Census Bureau [42], we could get the total populations of countries included. Combining coffee consumption data of them from International Coffee Organization in 2010 [43], we could calculate the coffee consumption per capita. We found that coffee consumption per capita (0.14 bags) among European countries included in our meta-analysis were twice greater than in the United States (0.07 bags), but did not observe an association between the consumption of coffee and risk of gastric cancer in European countries. In included original studies, we found that the average dose of coffee consumption of highest categories in those European populations is larger than in the United States. Conversely, it suggested that whether coffee consumption might have protective effect on the incidence of gastric cancer in some degree or not. The finding hints us that it is needed to further explore the dose–response association between coffee consumption and the risk of gastric cancer in future studies. In linear dose–response analysis, we found that the pooled RR of gastric cancer risk for an increase of coffee intake 2 cups per day was 0.98 (95 % CI, 0.85–1.13), which indicated that there were a null inverse association between coffee and gastric cancer. The null association might result from the limited included studies. We could preliminarily speculate that the larger difference between the groups of the highest and the lowest coffee consumption categories, the lower the risk estimates, if coffee had a protective effect on gastric cancer risk. Therefore, the lower risk estimates could be explained in a few categories of coffee consumption of our included studies. On the contrary, if coffee had some effect on the risk of gastric cancer we would expect the pooled RR to approach the null when the references group of coffee consumption includes coffee drinkers. According to available data, the pooled RR compared the highest with the lowest groups (including coffee drinkers) was 1.13 (95 % CI, 0.89–1.42), which was from 8 studies with 12 reports involving 1,205,876 participants and 2,418 patients with gastric cancer. Of course, this was likely to be an accidental finding. Additionally, we suspected that there is a U-shaped association between coffee consumption and gastric cancer risk in some extent. However, in nonlinear dose–response analysis, we did not observe any nonlinear association between coffee consumption and gastric cancer risk to date, which were likely limited by statistical power (nine studies comprising 803,500 participants). It is unclear whether the lack of a nonlinear dose–response association between coffee consumption and the risk of gastric cancer was due to potentially unfavorable effects of coffee at higher consumption levels or was because of residual confounding from other gastric cancer risk factors related to coffee consumption. More studies are warranted to investigate the potential difference between the different ethnic backgrounds.

Interestingly, another important finding was that coffee consumption increased the risk of gastric cancer in the group of equal to or less than 10 years follow-up, but did not have statistically significant association for more than 10 years follow-up. One possible explanation to this finding was that most studies have usually measured coffee consumption only once at the beginning of the study, and have assessed the outcomes at the end of the follow-up. Additionally, individuals frequently make changes to their diet, thus, observational studies investigating the association of baseline coffee consumption with gastric cancer risk cannot adequately account for mutative trends in intake. However, to our knowledge, no study has examined the association between changes in coffee and the risk of gastric cancer. We might preliminarily speculate that the longer the follow-up duration, the more people could change their coffee intake, which might weaken the association between coffee consumption and gastric cancer risk. The interesting finding deserves attention from related researchers. More studies investigating the association between changes in coffee consumption and gastric cancer over time are needed, which will help to explore the dose–response relationship with them.

Coffee is a complex mixture of over a thousand chemicals. Some constituents (such as very small amounts of aromatic hydrocarbons and heterocyclic amines) have been described as having genotoxic and mutagenic properties [11, 44]. For example, coffee contains acrylamide and caffeine, which have potential carcinogenic effects [45, 46]. In contrast, coffee contains many bioactive compounds including phenolic acids with strong antioxidant properties and cafestol and kahweol with anticarcinogenic activity [47]. Our findings provided an importantly open research thought for whether coffee consumption indeed had some degree protective effect on the incidence of gastric cancer or not. However, it lacked comprehensively biological mechanism research at the moment. Thus, both population and animal studies are needed to strengthen the exploration of biological mechanisms that link the coffee consumption and gastric cancer.

It is well acknowledged that the coffee bean types [48, 49], and roasting procedure [50] might affect coffee chemical composition [51]. We could preliminarily speculate that the difference conducting among geographic region might be that the type of coffee beans (Robusta versus Arabica), brewing methods (Instant, soluble, roast and ground), and caffeine content vary among ethnic groups and cultures. However, most of our included studies did not provide above detail information about coffee consumption characteristics.

Our study was unable to estimate the association between decaffeinated coffee and gastric cancer risk, because only one study separately reported the risk estimates between them. However, the study analyzing decaffeinated coffee consumption [39] found no relation with gastric cancer risk. It is not possible to separate the drinkers of both caffeinated and decaffeinated coffee from drinkers of decaffeinated coffee only. In general, drinkers consumed low amount of decaffeinated coffee [51].

### Strengths and limitations

Our review is very valuable and crucial though it is an updated meta-analysis. Firstly, we not only included the prospective cohort studies, also added 4 times as many participants as the previous review, which provided stronger and more reliable evidence. Secondly, on the basis of our subgroup analysis, a significantly positive association between coffee consumption and gastric cancer risk was observed in the United States populations, which provided a clue for future study of how the biological mechanisms of coffee consumption and gastric cancer are affected by ethnicity. Thirdly, we not only analyzed the association of higher coffee consumption with gastric cancer risk, but also conducted the dose–response analysis to evaluate the linear and non-linear relations by using all the categories of data, which could help to quantify the associations and examine the shape of these possible associations.

There are some limitations to this meta-analysis. Firstly, different methods of assessment were used in the included studies, and the units and cut-offs of coffee consumption were heterogeneous across different studies. Nevertheless, we used RRs for the highest versus the lowest category of coffee consumption, which could, to some extent, reduce the bias caused by different units. Secondly, the study relied on self-reported engagement in coffee consumption, which was likely to cause the misclassification of exposure, and may underestimate the reported associations. Thirdly, our meta-analysis did not take into consideration the differences of coffee bean types, brewing methods, and serving sizes for coffee among the included studies because of no sufficient information in the original studies.

Some suggestions should be considered in future research. Firstly, most of the studies included were conducted in the United States and Europe, while only two in Japan and one in Singapore. Given the underlying disease-effect discrepancy among different geographic locations and ethnicities, more studies should be conducted in other populations from Asian, African and South America. Secondly, in observational studies, investigators should pay attention to collect the information on the types of coffee bean, brewing methods, roasting procedure, preparation, cup size, and duration of use, which might allow more detailed analysis into the association between the characteristic of coffee consumption and gastric cancer risk. Thirdly, we should investigate the association of changes in coffee consumption with the occurrence of gastric cancer, and further accurately assess the association between coffee consumption and the risk of gastric cancer. Finally, coffee composition is very complex, thus, more population-based epidemiological investigations and animal experimental studies are needed to further explore the potential biological mechanisms link that the coffee intake and gastric cancer.

## Conclusions

In conclusion, the results of this meta-analysis do not support the hypothesis that higher coffee consumption is associated with gastric cancer risk, and do not suggest a nonlinear relationship between coffee consumption and gastric cancer risk. Subgroup analyses suggest a positive association between higher coffee consumption and risk of gastric cancer in the United States population and in the group of equal to or less than 10 years follow-up. Studies with larger sample size and longer follow-up time are warranted to confirm these subgroup results.

## Ethics approval

Ethical approval is not required for this review.

## Abbreviations

BMI: Body mass index
CI: Confidential interval
FFQ: Food frequency questionnaires
MOOSE: Meta-analysis of Observational Studies in Epidemiology
RR: Relative risk
